# The effects of dietary seaweed inclusion on growth performance of broiler chickens: a systematic review and meta-analysis

**DOI:** 10.12688/f1000research.25726.1

**Published:** 2020-09-03

**Authors:** Faizal Andri, Nanung Danar Dono, Heru Sasongko, Zuprizal Zuprizal

**Affiliations:** 1 Doctoral Program of Animal Science, Faculty of Animal Science, Universitas Gadjah Mada, Yogyakarta, 55281, Indonesia; 2 Department of Animal Nutrition and Feed Science, Faculty of Animal Science, Universitas Gadjah Mada, Yogyakarta, 55281, Indonesia; 3 Department of Animal Production, Faculty of Animal Science, Universitas Gadjah Mada, Yogyakarta, 55281, Indonesia

**Keywords:** alginate, body weight gain, fucoidan, fucoxanthin, functional feed, laminarin, macroalgae, poultry

## Abstract

**Background: **There has been great interest in the use of seaweed as a functional feed ingredient for poultry in the last decade. This study aimed to assess the effects of dietary seaweed inclusion on growth performance of broiler chickens by using a systematic review and meta-analysis approach.

**Methods: **A systematic search of published research articles related to seaweed, broiler chickens, and growth performance was conducted using three online databases (Scopus, PubMed, and SciELO). Mean values, standard deviation, and sample size were extracted from each eligible study. The estimated effect size was then quantified using Hedges’ *g* with a 95% confidence interval (CI). Data were pooled using a fixed-effect model due to the absence of heterogeneity after being pre-checked using the *I* ^2^ statistic.

**Results: **A total of six studies (nine comparisons) involving 2,257 broiler chickens were accommodated in this study. The seaweed type consisted of seaweed blend, *Laminaria japonica*, *Undaria pinnatifida*, *Hizikia fusiformis*, and *Ulva lactuca*. The inclusion dose ranged from 2 to 30 g/kg, while the intervention duration ranged from 21 to 42 days. No substantial heterogeneity among studies ( *I*^2^ = 0.00%) was found for feed intake, body weight gain, and feed conversion ratio. Dietary seaweed had no significant effect on feed intake (Hedges’ *g* = 0.19; 95% CI = -0.22 to 0.60; *P* = 0.280). However, broiler chickens fed dietary seaweed had superior body weight gain (Hedges’ *g* = 0.64; 95% CI = 0.22 to 1.06; *P* = 0.000) and preferable feed conversion ratio (Hedges’ *g* = -0.53; 95% CI = -0.95 to -0.11; *P* = 0.004).

**Conclusions: **The current investigation highlights that dietary seaweed had growth-promoting potency for broiler chickens. However, more research on this issue is still required to build more comprehensive evidence.

## Introduction

There has been great interest in the use of seaweed as a functional feed ingredient for poultry in the last decade. The primary functional compounds in seaweed are polysaccharides, peptides, fatty acids, phlorotannins, and carotenoids ^1– 3^. These compounds have antimicrobial, antioxidant, and immunomodulatory properties ^4– 7^, which are essential to support production performance.

Several reviews have compiled studies regarding the effect of dietary seaweed inclusion on poultry performance ^8– 13^. However, those reviews were based on a narrative approach, which mostly led to an inconclusive epilogue due to the contradictory results among studies. The use of systematic review and meta-analysis has become popular in animal science ^14– 18^. This methodology can integrate and determine the overall effect of interventions from several studies to provide more accurate insight than the narrative review. Therefore, this study aimed to assess the effect of dietary seaweed inclusion on the growth performance of broiler chickens using a systematic review and meta-analysis approach.

## Methods

This study was reported based on the Preferred Reporting Items for Systematic Reviews and Meta-Analyses (PRISMA) guidelines ^19^. The PRISMA checklist is presented in *Reporting guidelines* ^20^.

### Eligibility criteria

Research articles published in peer-reviewed journals between the years of 2000 to 2020 and written in English were eligible. Additionally, eligible studies also should fulfill the participants, interventions, comparisons, outcomes, and study design (PICOS) criteria given in Table 1.

**Table 1.** PICOS criteria.

Items	Criteria
Participants	Broiler chickens
Interventions	Inclusion of dietary seaweed either as such or fermented product
Comparisons	Diet without seaweed inclusion (control)
Outcomes	Feed intake, body weight gain, and feed conversion ratio
Study design	Controlled trials

### Searching strategy

The online search was conducted using three databases, namely Scopus, PubMed, and SciELO, with the queries in Table 2. The final search was on 25 June 2020. The references from the included studies were also screened to find additional eligible studies.

**Table 2.** The search query in Scopus, PubMed, and SciELO databases.

Database	Search query
Scopus	(TITLE-ABS-KEY (seaweed OR macroalgae) AND TITLE-ABS-KEY (growth OR performance) AND TITLE-ABS-KEY (broiler OR chicken))
PubMed	((seaweed[Title/Abstract] OR macroalgae[Title/Abstract]) AND (growth[Title/Abstract] OR performance[Title/Abstract])) AND (broiler[Title/Abstract] OR chicken[Title/Abstract])
SciELO	(ab:(seaweed OR macroalgae)) AND (ab:(growth OR performance)) AND (ab:(broiler OR chicken))

### Study selection

Firstly, the duplicate reports were removed from the database in Microsoft Excel for Microsoft 365 software. After that, the title and abstract were examined. Irrelevant studies, non-English reports, and review articles were then excluded from the list. The full text was further evaluated according to the eligibility criteria.

### Data collection

Mean values, standard deviations, and sample sizes were extracted from each included study. The target variables in this study were feed intake (FI), body weight gain (BWG), and feed conversion ratio (FCR). When a study used the standard error of means as a variance measure, it was converted into standard deviation ^21^. In the case of more than one seaweed type used in a study, each treatment was coded individually. On the other hand, the treatment was pooled when a study used more than one dose of the same seaweed type ^22^. None of the authors were contacted for further clarification.

### Data analysis

Data analysis was performed using Meta-Essential version 1.5 ^23^. The estimated effect size (the difference between seaweed intervention and control) was quantified using Hedges’ *g* with a 95% confidence interval (CI) ^24^. Data were pooled using a fixed-effect model due to the absence of heterogeneity after being pre-checked using the *I* ^2^ statistic ^25^. A significant effect was declared when the overall estimated effect size had *P* < 0.05. Publication bias was not evaluated because the number of the included studies was fewer than 10 ^26^.

## Results

**Figure 1.**
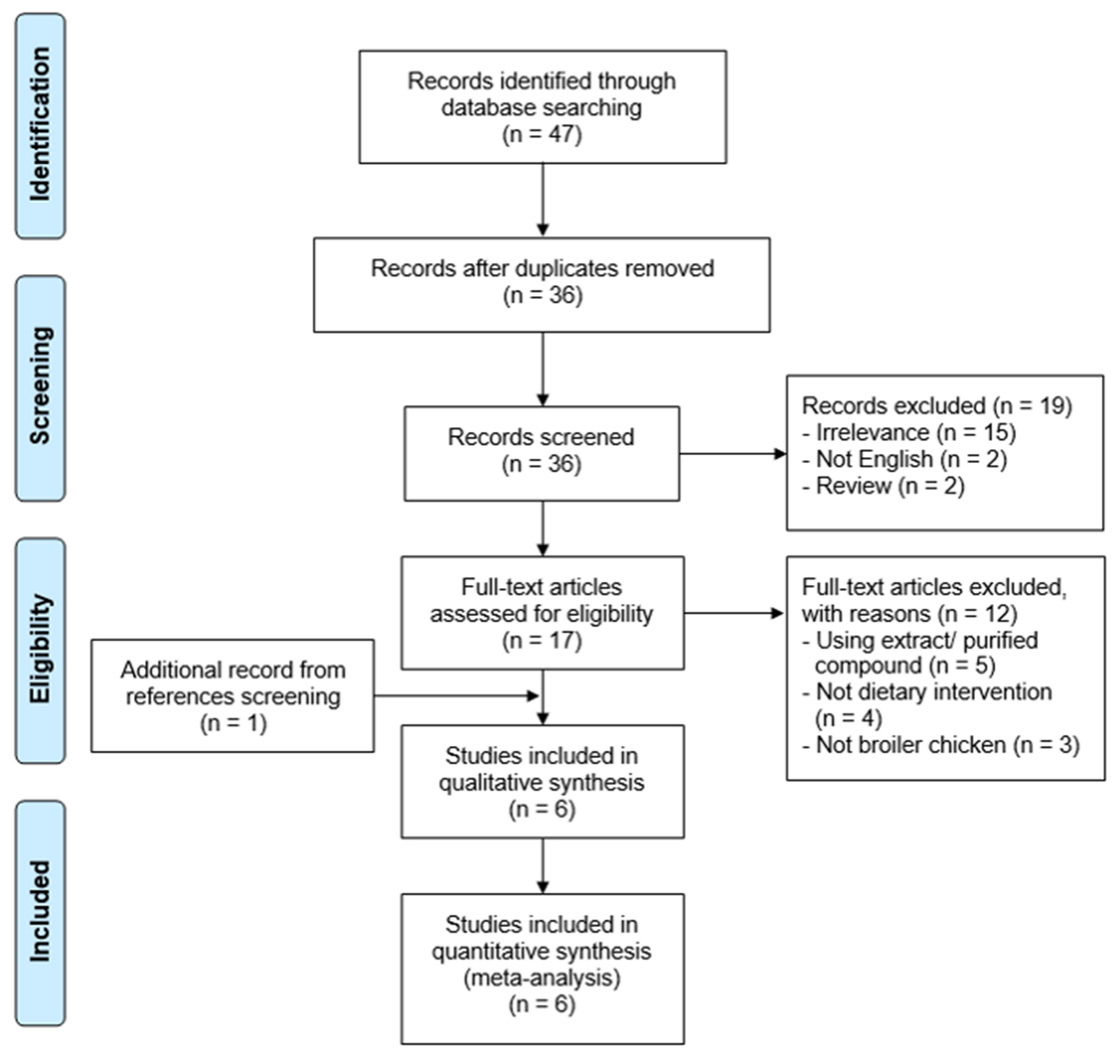
PRISMA flow diagram.

The PRISMA flow diagram is shown in Figure 1. The search using three online databases identified 47 records. Of these, five studies met the eligibility criteria. Additionally, one study from reference screening also found to be eligible. Therefore, a total of six studies, with nine comparisons were included in the synthesis.

The details of the included studies are shown in Table 3. A total of 2,257 broiler chickens were involved in this study. The seaweed type used included seaweed blend ^27^, *Laminaria japonica* ^28, 30^, *Undaria pinnatifida* ^29, 31^, *Hizikia fusiformis* ^31^, and *Ulva lactuca* ^32^. The inclusion dose ranged from 2 to 30 g/kg, while the intervention duration ranged from 21 to 42 days. The extracted data of target variables is presented as *Extended data* ^33^.

**Table 3.** Details of the included studies.

Study name	N	Strain	Sex	Diet type	Seaweed type	Dose (g/kg)	Period (d)
Mohammadigheisar *et al*. ^27^	864	Ross	Male	Corn-SBM	Blend of brown, green, and red seaweed	5, 10, and 20	1-42
Bai *et al*. ^28^	144	Arbor Acres	Mixed	Corn-SBM	*L. japonica*	10	1-42
Shi *et al*. ^29^	384	Ross	Mixed	Corn-SBM	Fermented *U. pinnatiﬁda*	2	1-35
Ahmed *et al*. ^30^	70	Ross	Mixed	Corn-SBM	Fermented *L. japonica*	5	1-35
Choi *et al*. ^31^	750	Ross	Male	Corn-SBM	*U. pinnatiﬁda* (as such and fermented) and *H. fusiformis* (as such and fermented)	5	1-35
Abudabos *et al*. ^32^	45	Ross	Male	Corn-SBM	*U. lactuca*	10 and 30	12-33

n: number of broiler chickens, SBM: soybean meal.

**Figure 2.**
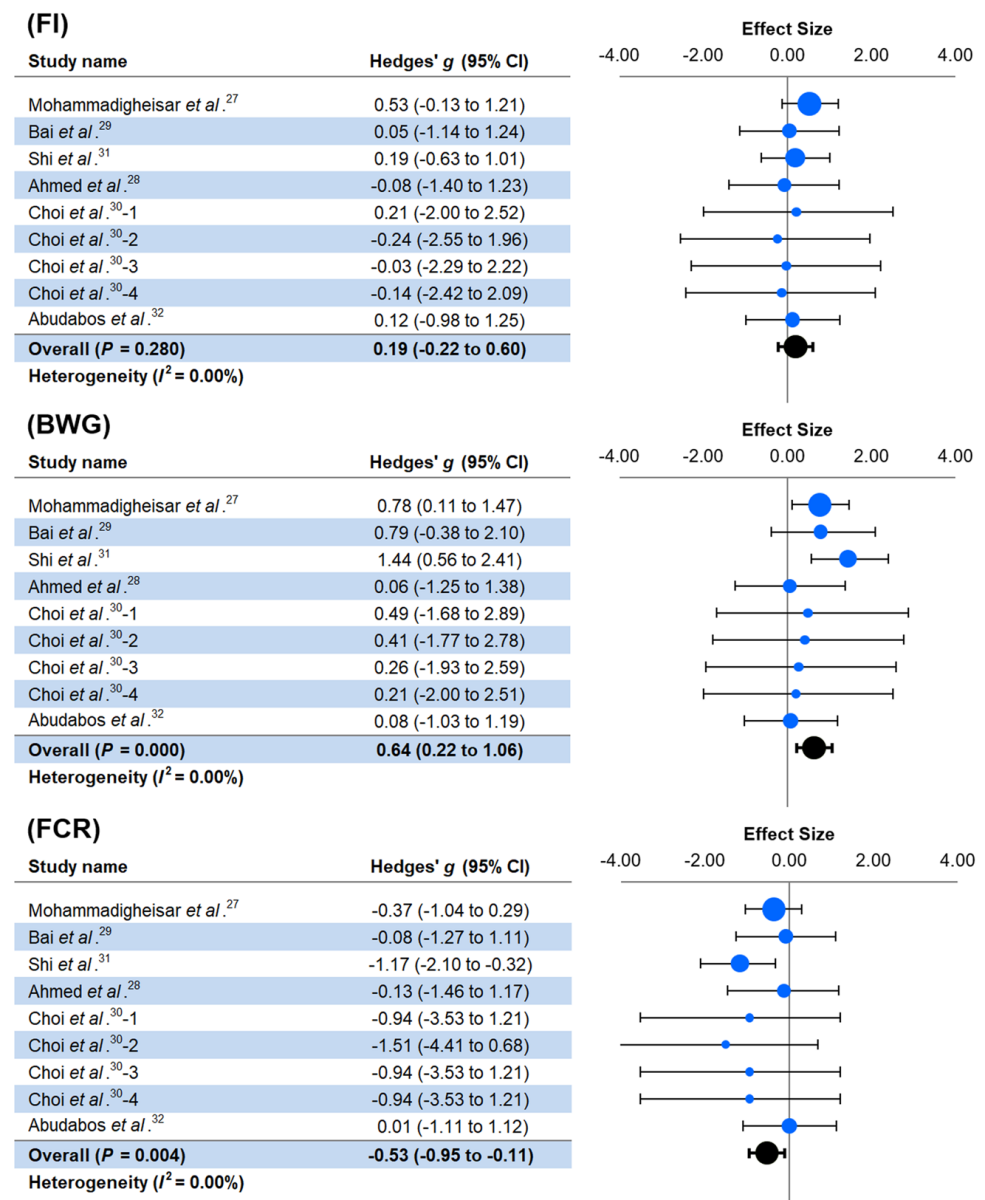
Forest plot showing the effect of dietary seaweed inclusion on growth performance of broiler chicken. FI: feed intake, BWG: body weight gain, FCR: feed conversion ratio, CI: confidence interval.

As shown in Figure 2, no substantial heterogeneity was found for any variables ( *I* ^2^ = 0.00%). Dietary seaweed had no significant effect ( *P* > 0.05) on FI. However, this intervention significantly improves ( *P* < 0.05) the BWG and FCR of broiler chickens. The overall estimated effect size values for BWG and FCR were 0.64 and -0.53, respectively, which were equivalent to the raw mean difference of 77.24 g and -0.07, respectively.

## Discussion

In this study, the use of dietary seaweed had a beneficial impact on BWG and FCR of broiler chickens. According to Cohen ^34^, the overall estimated effect size of BWG and FCR in the present study was categorized into the medium (0.5) to large (0.8) standardized effect size. In agreement with this finding, other studies also showed that the use of seaweed could improve production performance in laying hens ^35– 37^ and geese ^38^. Seaweed contained numerous unique bioactive substances such as alginate, ulvan, laminarin, fucoidan, and fucoxanthin. Those compounds could inhibit the colonization of pathogenic bacteria ( *Escherichia coli* and *Salmonella* Enteritidis), promote the growth of beneficial gut microbes (lactic acid bacteria), improve small intestinal architecture, antioxidant status, and immune response ^39– 43^. Together, those mechanisms could ultimately improve the growth performance of broiler chickens.

Nevertheless, this finding is accompanied by the limited number of included studies. It is possible that not all relevant studies were captured by the searching strategies. For those reasons, the current results should be elucidated with caution. Moreover, due to the enormous diversity of seaweed in nature (around twenty thousand species) ^44^, future studies regarding seaweed intervention in broiler chickens are still open and strongly encouraged to provide a robust body of knowledge.

## Conclusions

The current systematic review and meta-analysis highlight that dietary seaweed had no adverse effect on FI. Instead, they could improve BWG and FCR of broiler chickens. However, more research on this issue is still required to build more comprehensive evidence.

## Data availability

### Underlying data

All data underlying the results are available as part of the article and no additional source data are required.

### Extended data

Figshare: Extended data for ‘The effects of dietary seaweed inclusion on growth performance of broiler chickens: a systematic review and meta-analysis’. https://doi.org/10.6084/m9.figshare.12721454.v1 ^33^.

This project contains the following extended data in DOC format:

- Extended data 1 – extracted data of feed intake
- Extended data 2 – extracted data of body weight gain
- Extended data 3 – extracted data of feed conversion ratio
- Extended data 4 – list of included studies

### Reporting guidelines

Figshare: PRISMA checklist for 'The effect of dietary seaweed inclusion on growth performance of broiler chickens: a systematic review and meta-analysis'. https://doi.org/10.6084/m9.figshare.12721118.v1 ^20^.

Data are available under the terms of the Creative Commons Zero “No rights reserved” data waiver (CC0 1.0 Public domain dedication).
