# Supplementary material for: The effects of dietary seaweed inclusion on growth performance of broiler chickens: a systematic review and meta-analysis
Source: F1000Res. 2020 Sep 3;9:1087. [Version 1] doi: 10.12688/f1000research.25726.1 (PMC8422340; doi:10.12688/f1000research.25726.1)
Supplement: Peer review history

# Peer review: Reviewer response for version 1

**DOI:** 10.5256/f1000research.28391.r92551

**Published:** 2021-09-06

**Authors:** Henny Akit

**Affiliations:** 1 Department of Animal Science, Faculty of Agriculture, Universiti Putra Malaysia, Serdang, Malaysia

Some of the keywords such as alginate, fucoidan, fucoxanthin and laminarin were not mentioned in the abstract and introduction. You may consider excluding these keywords.

 The rationale for the systematic review was to provide a more accurate insight than the narrative review. The authors may add on how this insight could benefit researchers or related feed industries for seaweed application. 

 In discussion, the authors explained the possible mechanisms that led to improved broiler performance. I suggest that the authors include the year of broiler studies used. In addition, I think more points should be included emphasising how the results could benefit other researchers, for example, suggestions on the inclusion rate of seaweed, etc. Since this is a review paper, I feel that the discussion should be more extensive.

Are the rationale for, and objectives of, the Systematic Review clearly stated?

Yes

Is the statistical analysis and its interpretation appropriate?

I cannot comment. A qualified statistician is required.

Are sufficient details of the methods and analysis provided to allow replication by others?

Yes

Are the conclusions drawn adequately supported by the results presented in the review?

Yes

Reviewer Expertise:

I am an expert in broiler nutrition currently doing research in alternative protein ingredients in broilers. However, i am not an expert in the statistic part.

I confirm that I have read this submission and believe that I have an appropriate level of expertise to confirm that it is of an acceptable scientific standard, however I have significant reservations, as outlined above.

# Peer review: Reviewer response for version 1

**DOI:** 10.5256/f1000research.28391.r70759

**Published:** 2020-11-05

**Authors:** Mary Kivali Ambula

**Affiliations:** 1 Department of Animal Sciences, Egerton University, Njoro, Kenya

- The keywords listed are not mentioned much in the text which might make searching for the article a little difficult.
- Clearly state if seaweed was used as a substitute for another ingredient or not.
- Conclusions may not fully be drawn from the results since information on the type of basic diets is not provided e.g were they typical corn-soybean broiler diets or what?
- The effect of seaweed on broilers may also depend on other feed ingredients used (alfalfa meal, corn gluten meal etc). Authors can clarify this to the reader to inform further research.

Are the rationale for, and objectives of, the Systematic Review clearly stated?

Yes

Is the statistical analysis and its interpretation appropriate?

Yes

Are sufficient details of the methods and analysis provided to allow replication by others?

Yes

Are the conclusions drawn adequately supported by the results presented in the review?

Partly

Reviewer Expertise:

Poultry nutritionist with interest in use of non-conventional feed ingredients in chicken diets

I confirm that I have read this submission and believe that I have an appropriate level of expertise to confirm that it is of an acceptable scientific standard.

# Peer review: Reviewer response for version 1

**DOI:** 10.5256/f1000research.28391.r70757

**Published:** 2020-09-28

**Authors:** Samadi Samadi

**Affiliations:** 1 Animal Husbandry Department, The Faculty of Agriculture, Syiah Kuala University, Banda Aceh, Indonesia

This manuscript aims to assess the effects of dietary seaweed inclusion on the growth performance of broiler chickens by using a systematic review and meta-analysis approach. The type of work is suitable for publication in the journal. However, the manuscript needs some correction before it can be fully accepted. The comments are as follows.

- Introduction was too short and needs more explanation from the previous study (state of arts) relating to seaweed on broiler production.
- From PRISMA flow diagram, there were a lot of studies excluded for data analysis, out of 47 studies only 6 studies were continued for quantitative analysis. Is the data sufficient to make conclusions for this meta-analysis study?
- There was a wide range of seaweed inclusion in broiler performance from the data (2-30 g/kg). Any explanation?
- Discussion of this study was too short, it is better to extend more explanation regarding to the findings based on this study.
- Based on the literature, are there any different bioactive compound from various seaweed in nature (around twenty thousand species) #44
- Conclusion of this study was too general, are there any recommendations for seaweed inclusion in the broiler diet based on the meta-analysis data, since the seaweed concentration from the literature of this study was too wide-ranging from 2-30 g/kg diet.

I suggest it is better also to add one more table informing about FI, BWG, and FCR from 6 studies to get information for the readers about the broiler performance data.

Are the rationale for, and objectives of, the Systematic Review clearly stated?

Partly

Is the statistical analysis and its interpretation appropriate?

Yes

Are sufficient details of the methods and analysis provided to allow replication by others?

Partly

Are the conclusions drawn adequately supported by the results presented in the review?

Partly

Reviewer Expertise:

Animal Nutrition

I confirm that I have read this submission and believe that I have an appropriate level of expertise to confirm that it is of an acceptable scientific standard, however I have significant reservations, as outlined above.
